# Verifying causes of death in Thailand: rationale and methods for empirical investigation

**DOI:** 10.1186/1478-7954-8-11

**Published:** 2010-05-18

**Authors:** Chalapati Rao, Yawarat Porapakkham, Junya Pattaraarchachai, Warangkana Polprasert, Narumol Swampunyalert, Alan D Lopez

**Affiliations:** 1 School of Population Health, University of Queensland, Brisbane, Australia; 2 Ministry of Public Health, Bangkok, Thailand; 3 Department of Community Medicine, Thammasat University, Pathumthani, Thailand; 4 School of Health Sciences, Sukhothaithummathirat Open University, Bangkok, Thailand; 5 Health Information Systems Knowledge Hub, University of Queensland, Brisbane, Australia

## Abstract

**Background:**

Cause-specific mortality statistics by age and sex are primary evidence for epidemiological research and health policy. Annual mortality statistics from vital registration systems in Thailand are of limited utility because about 40% of deaths are registered with unknown or nonspecific causes. This paper reports the rationale, methods, and broad results from a comprehensive study to verify registered causes in Thailand.

**Methods:**

A nationally representative sample of 11,984 deaths was selected using a multistage stratified cluster sampling approach, distributed across 28 districts located in nine provinces of Thailand. Registered causes were verified through medical record review for deaths in hospitals and standard verbal autopsy procedures for deaths outside hospitals, the results of which were used to measure validity and reliability of registration data. Study findings were used to develop descriptive estimates of cause-specific mortality by age and sex in Thailand.

**Results:**

Causes of death were verified for a total of 9,644 deaths in the study sample, comprised of 3,316 deaths in hospitals and 6,328 deaths outside hospitals. Field studies yielded specific diagnoses in almost all deaths in the sample originally assigned an ill-defined cause of death at registration. Study findings suggest that the leading causes of death in Thailand among males are stroke (9.4%); transport accidents (8.1%); HIV/AIDS (7.9%); ischemic heart diseases (6.4%); and chronic obstructive lung diseases (5.7%). Among females, the leading causes are stroke (11.3%); diabetes (8%); ischemic heart disease (7.5%); HIV/AIDS (5.7%); and renal diseases (4%).

**Conclusions:**

Empirical investigation of registered causes of death in the study sample yielded adequate information to enable estimation of cause-specific mortality patterns in Thailand. These findings will inform burden of disease estimation and economic evaluation of health policy choices in the country. The development and implementation of research methods in this study will contribute to improvements in the quality of annual mortality statistics in Thailand. Similar research is recommended for other countries where the quality of mortality statistics is poor.

## Introduction

Reliable information on levels of mortality and leading causes of death is essential to guide priorities for resource allocation within the health sector in order to increase longevity and improve quality of life. In combination with measures of disease or condition-specific morbidity, these data are also useful in monitoring the epidemiological impact of specific health interventions or broader health programs as well as their cost-effectiveness, applying the burden of disease approach [1]. However, such evidence-based health development strategies are feasible only when reliable and timely epidemiological data are available for countries at national and subnational levels. Mortality statistics are fundamental to such priority-setting approaches in public health policy and planning. Recent assessments of global health statistics have suggested that only about one-third of all countries have functional national civil registration systems, which are the optimal source for mortality data [2]. Much needs to be done to rectify this situation through strategic approaches to improve the availability and quality of mortality statistics [3].

Thailand is among another group of about one-third of the world's countries that produce population-level mortality statistics from civil registration, but that are of limited utility owing to problems with data quality [4]. These limitations severely hinder the potential use of these data for epidemiological assessments or health development strategies. Over the past two decades, Thailand has introduced several reforms to improve its national civil registration and vital statistics systems, described in detail elsewhere [5]. However, the reliability and validity of registered causes of death should also be periodically assessed to guide the utility of available vital statistics for health policy and planning. Therefore, a comprehensive field research study was conducted in Thailand during 2005-2008 to verify and evaluate the quality of cause-of-death attribution in a nationally representative sample of nearly 10,000 deaths that occurred in 2005.

We report the rationale, methods, findings, and implications of the study in this series of articles. Starting with an overview of the background, rationale, and objectives of the study, this first paper then describes the overall design, sampling strategy, and broad principles of data collection and analysis. The next two articles describe the detailed methods and findings from the two arms of the study, respectively covering deaths in the sample that had occurred in hospitals and deaths that occurred at home or elsewhere [6,7]. A fourth article applies the results from the field studies to adjust identified biases in registration data and derive an overall estimate of cause-specific mortality for Thailand in 2005 [8]. These adjusted mortality statistics form the primary evidence base for burden of disease assessment, economic evaluation, and the identification of national health policy priorities in Thailand.

### Civil registration and vital statistics in Thailand

A brief overview of civil registration and vital statistics in Thailand helps place the research study into context. Birth and death registration in Thailand commenced in 1916, supplemented by the introduction of the household register in 1956, a copy of which is issued to the household as proof of civil status [9]. In 1991, a revised Civil Registration Act nominated the Bureau of Registration Administration, Ministry of Interior, as the central agency responsible for civil registration through a network of offices at local, district, municipality, and provincial levels and stipulated the requirement for death notification within 24 hours [10]. Causes of deaths in hospitals are recorded using a Thai version of the standard International Form of Medical Certificate of Causes of Death, with an additional column in which the certifying physician records one cause in Thai language to be used for registration purposes. It is this cause that is entered in the national registration electronic database. For unnatural (i.e., injury) deaths, causes are certified following forensic investigation by a physician using a similar process. For deaths occurring outside hospitals (about 65% of all deaths), local registrars inquire about the cause of death from family members and also seek documentary evidence, wherever possible, from previous hospitalization or medical attention during the illness preceding death. Local registrars then record the reported cause of death in Thai. After computerization, a complete extract of the database (with a single cause in Thai for each death) is transferred to the Ministry of Public Health, where the causes are coded according to ICD-10 and tabulated by age, sex, and cause for subsequent dissemination and analysis [5].

### Characteristics of Thai death registration data

Thailand is one of the few Asian countries that have submitted data on a regular basis to the World Health Organization since 1950 [2]. A recent assessment of mortality statistics in Thailand identified that the sound legal framework and institutional capacity for implementation are strengths that result in regular and timely data [5]. Computerization of civil registration over the past two decades has vastly improved efficiency of registration services as well as compilation of statistics. However, mortality data are limited by the incompleteness of death registration and more so by the high proportion of ill-defined causes of death [5]. These limitations also preclude the application of detailed technical criteria to assess additional aspects of data quality in terms of validity and reliability [11].

In recent years, a gradual increase in the number of registered deaths has been noted, from 264,350 in 1991 to 395,374 in 2005 [12]. This increase is attributable to various factors, including the 1991 revisions to the legal framework, administrative reforms to registration procedures, and computerized compilation of data, all of which came into effect over the period up to 1996 [10]. However, the current level of completeness of adult death registration remains a subject of ongoing research, with the application of different demographic methods yielding completeness estimates ranging from 80% to 95% [13,15]. While there is general consensus regarding the extent of under-registration of child deaths (approximately 50%) [5,10,13,16], the overall completeness of death registration in Thailand remains uncertain.

In terms of recorded causes of death, about 40% are routinely coded to symptoms, signs, and ill-defined conditions [17]. The bulk of these ill-defined deaths occur outside health facilities, although in-hospital cause-of-death attribution is also problematic, arising from the process of recording the single cause of death for registration in Thai language. Hence, the validity of registered causes of death is questionable and greatly limits their public health utility. A study conducted by Boonthai et al found low validity of diagnoses on death certificates issued in a sample of hospital deaths during the period 1967-1984 [18]. Choprapawon and colleagues later conducted a detailed verification of 47,632 deaths that occurred in 15 provinces in Thailand during 1997-1999, using a combination of verbal autopsy interviews and medical record review, as applicable [10]. Their findings indicated poor validity of registered causes for deaths in hospitals as well as for deaths occurring at home.

### Rationale for current research

In view of the existing problems with cause-of-death ascertainment, further reforms to the Thai death registration system were pilot-tested in 18 provinces from 2001 to 2003. Direct support from the medical profession was sought to certify causes for nonhospital deaths, using information from previous contact with health services and/or limited verbal autopsy interviews [10]. However, potential medico-legal implications from attributing causes without having attended to the deceased restricted the implementation of this reform by the medical profession [5].

Given these circumstances, the Thai Ministry of Public Health proposed a long-term strategy to improve cause-of-death statistics using two approaches [19]. The ministry proposed that for deaths in hospitals, capacity should be strengthened to improve accuracy of medical certification of cause of death as well as selection and coding of underlying causes of death according to ICD principles. For home deaths, local health personnel should be trained in the use of available medical records and verbal autopsy procedures to improve accuracy in the recording of causes of death at registration. As an interim measure, the Thai Ministry of Health identified the need to conduct research studies to verify causes in a national sample of registered deaths every five years and to use this research to derive periodic estimates of cause-specific mortality patterns to monitor health status and inform health policy and planning in Thailand [5,19]. This set of articles describes the methods and findings from such research on a sample of deaths that occurred in 2005. This study forms the basis for a broader study to estimate the relative cost-effectiveness of various intervention choices in Thailand for which corrected age-, sex-, and cause-specific death rates were required to more reliably estimate the burden of disease applicable to each intervention [20].

### Study objectives

The overall goal of the study was to derive the best estimate of cause-specific mortality patterns in Thailand for 2005. Specific objectives were to:

1. Ascertain, certify, and code underlying causes of death according to ICD-10 principles for a sample of deaths that occurred in hospitals through medical record review.

2. Develop, test, and implement standard verbal autopsy procedures (adapted to the Thai setting) for ascertainment of the probable underlying causes for deaths in the study sample that had occurred outside hospitals.

3. Utilize findings from this research to adjust vital registration data and derive best estimates of age-, sex-, and cause-specific mortality rates in Thailand for 2005.

In addition, it was anticipated that the research activity would also accomplish a longer-term outcome, namely building capacity among Thai health professionals (physicians, paramedical staff, biostatisticians, and epidemiologists) to critically assess vital statistics data and improve the quality of causes of death recorded at registration in Thailand.

## Methods

### Study design

A cross-sectional study was designed to verify causes of death for a nationally representative, multistage stratified cluster sample of deaths that occurred in Thailand during 2005. The sample was drawn from the mortality database maintained by the Bureau of Policy and Strategy, Ministry of Public Health [12]. The sampling unit was a registered death of a Thai citizen, identified by assigned national identification number, who was a permanent resident in one of the sample provinces included in the study. The cause or causes for each study death were investigated through verbal autopsy (VA). For study deaths that had occurred in a hospital, relevant medical records were accessed and reviewed to derive reference diagnoses. These reference diagnoses were used to validate and correct registered causes in the study sample, as well as to assess the validation characteristics of VA procedures for individual causes of death. For deaths that had occurred at home, VA diagnoses were used to estimate cause-specific mortality patterns, with subsequent adjustments of biases for individual causes of death, as observed from the VA validation component. Proportionate mortality distributions for health facility deaths in the study sample were used to estimate mortality patterns for all deaths in hospitals in Thailand (about 140,000 deaths each year). Similarly, VA-based proportionate mortality distributions for deaths outside hospitals were used to estimate mortality patterns for such deaths for 2005 (about 254,000 deaths). The overall proportionate (by cause) mortality estimates from the two settings were then applied to the national estimate of total mortality derived from demographic analyses to obtain corrected national cause-specific mortality estimates by age and sex for Thailand in 2005.

### Issues in determining sample size

Several considerations influence sample size estimation for a study of this nature. While statistical considerations are paramount when computing sample size, the ultimate choice of such parameters is guided by available financial and human resources as well as time constraints. Also, epidemiological considerations influence the inclusion of important causes of death in the study. Such logistical and epidemiological considerations usually limit the number of causes of interest to a selection of leading causes of death.

In this study, generalizability of findings is essential to accomplish the overall objective to derive cause-specific mortality estimates for Thailand. Intuitively, a close fit between the proportionate distributions of causes of death in the registration data and the study sample would support generalizability. The 20 leading causes of death in the Thai registration data (based on the WHO Mortality Tabulation List 1 [21]) accounted for more than 85% of all deaths, including 38.2% from the leading registered cause of death--"symptoms, signs, and ill-defined conditions." Therefore, we chose a sample of deaths that matched the proportionate distribution for the 20 leading causes in the registration data.

The proportion of deaths from the 21st leading cause of death in the Thai registration data was 0.0118. We assumed that the proportionate distribution by cause in a random sample of deaths adequate to measure the proportion of deaths from this 21st leading cause (within defined statistical parameters of error and significance) is likely to match the proportionate distribution for the 20 leading causes of death in the sampling frame. Therefore, we used this proportion for the 21st leading cause to compute the study sample size (n) as follows:

where π = 0.0118, within a selected margin of error *d *= 0.0025%, at the 95% level of confidence. This suggested that a random sample of 7,168 deaths would be required to accurately measure the proportion of the 21st leading cause in registration data within the given margin of error and level of confidence, based on prior information as to the proportion of interest. Further, since this sample was designed to be implemented in clusters for logistical and operational reasons, a design effect of 1.4 was applied, yielding an overall sample size of just over 10,000 deaths (10,035) [22,23].

### Sampling plan

**Figure 1:**
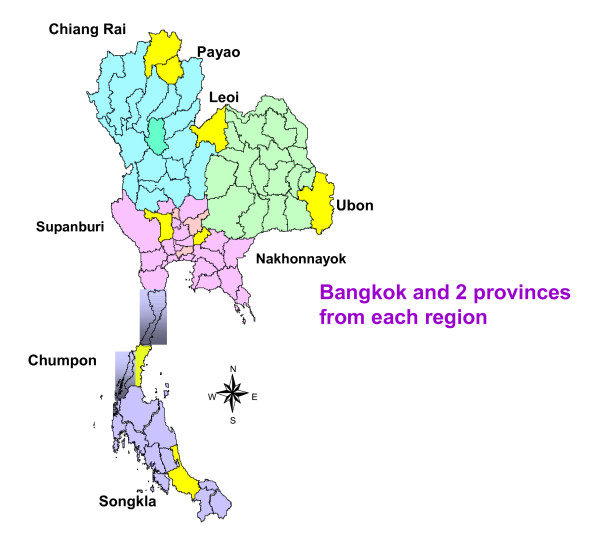
**Distribution of study provinces in Thailand**.

The nationally representative study sample was selected from the national death registration database using a multistage stratified clustered approach. At the first stage, Thailand was stratified into four broad regions - Northeast, North, Central, and South--as well as Bangkok, with the total sample of 10,000 deaths being distributed across regions according to the proportion of deaths from each region in the national death registration data. Each of the four regional samples was inflated by 15%, and by 50% for Bangkok, to account for potential losses to follow up, as suggested by the pilot study for this research and earlier research in Bangkok [10]. Subsequently, in each of the four broad regions, provinces were ordered according to numbers of registered deaths in 2005 and divided into two strata at the 50^th ^percentile. One province was randomly selected from each stratum, leading to two study provinces from each of the four broad regions. The adjusted (inflated) regional sample was then distributed between the two provinces proportionate to the number of deaths registered in 2005. Figure 1 shows the geographical distribution of the study provinces in Thailand.

In each study province, districts were ranked according to the number of registered deaths in 2005 and similarly divided into two strata at the 50^th ^percentile. In order to ensure representation of predominantly urban or rural communities as well as differential access to health facilities, districts within the provincial study sample were selected according to probability proportionate to size. In each stratum, a district was randomly selected at first, and the stratum-specific sample was proportionately allocated to the selected district according to probability proportionate to the number of deaths registered in 2005. Additional districts were selected one at a time with similar allocation of the study sample, cumulating the sample across selected districts until the province- stratum sample was attained. Within each selected district, the study deaths were randomly selected without replacement from all deaths registered during 2005.

For Bangkok, the study sample was distributed across the inner-, middle-, and outer-concentric zones of the Bangkok metropolitan area, and one district was randomly selected from each zone for inclusion in the study. The district-specific samples were determined according to probability proportionate to size, and within each district, the study sample was selected without replacement. Based on this strategy, a total of 11,984 deaths were selected into the study, distributed across 25 districts located in eight provinces from among the four broad regions of Thailand, and three districts from Bangkok.

### Data collection and processing

For each sampled death, relevant information on the age, sex, and identity of the deceased, date of death, as well as the address and the underlying cause of death recorded at registration (the VR cause of death) was extracted from the national death registration database for 2005. Subsequently, an initial visit was undertaken to the households of each of the deceased, to:

• confirm the address and the place of death of the deceased.

• set up an appointment for a verbal autopsy inquiry into the cause of death.

• obtain informed consent to access medical records to ascertain the cause of death in cases in which the death occurred in a health facility.

Subsequent data collection proceeded as follows:

1. For deaths in health facilities, in addition to the VR cause of death, an underlying cause was ascertained from a review of medical records, if available (the MR cause of death), as well as from an independent verbal autopsy (the VA cause of death).

2. For deaths at home, underlying causes were available from VR and from VA.

**Figure 2:**
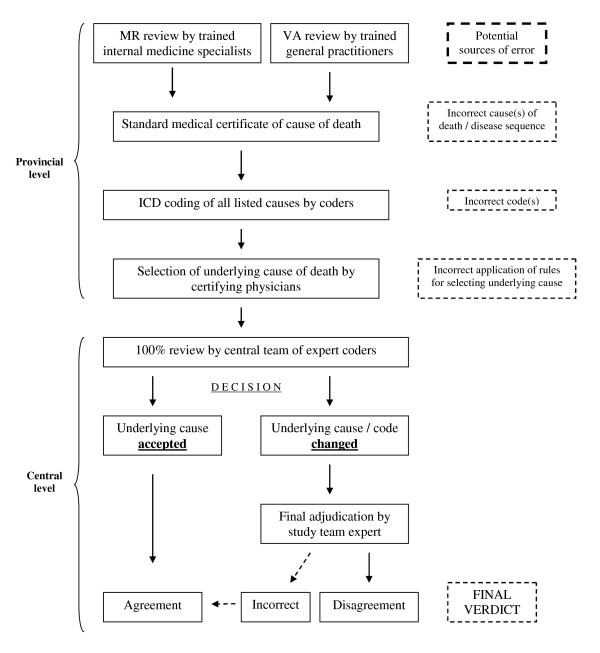
**Data processing and quality control measures for selection and coding of underlying causes of death in the study sample**. Incorrect = Change in ICD code at 3 or 4 character level, but no change at Mortality Tabulation List 1 level of aggregation. Disagreement = Change in ICD code resulting in change at Mortality Tabulation List 1 level of aggregation.

In a subsample of 2,232 hospital deaths, an audit procedure was conducted to assess the quality of information recorded on medical certificates of cause of death. Detailed data collection procedures, relevant training support, data processing, and management protocols are described in the separate articles for each arm of the study, along with key findings and their implications [6,8]. A detailed quality-control mechanism was implemented to ensure accuracy in the process of selection and coding of underlying causes of death (Figure 2). Measures were instituted to detect and correct errors in this process from a range of potential sources, and summary indices were developed to assess the overall quality of cause-of-death certification and ICD coding from either medical records review [6] or verbal autopsy questionnaires [7].

Underlying causes of death were derived for each death in the sample and classified according to the 10th revision of the International Classification of Diseases and Health Related Problems (ICD-10). These data were then aggregated to the ICD-10 Mortality Tabulation List 1 consisting of 103 cause categories, and all subsequent descriptive and comparative analyses were conducted using these aggregated data.

### Data analysis

**Figure 3:**
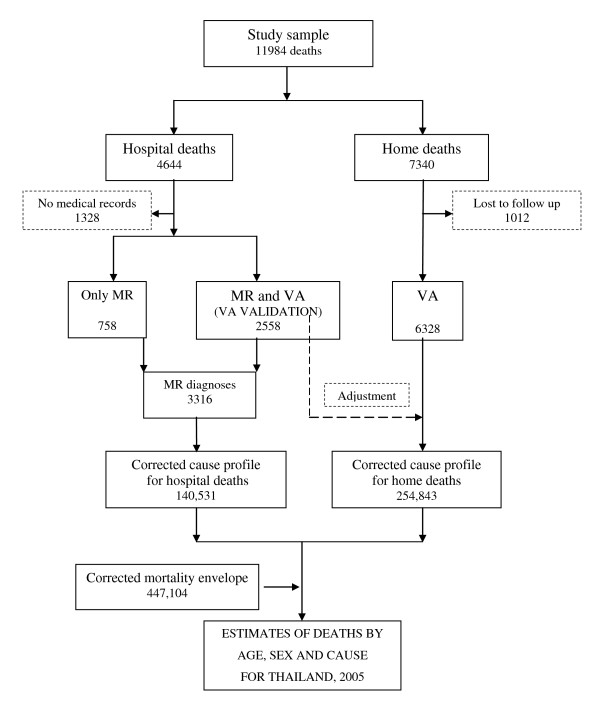
**Analytical plan for estimating cause-specific mortality in Thailand, 2005**.

Figure 3 describes the different elements of data analyses pertaining to each arm of the study and the process used to derive cause-specific mortality estimates for Thailand using the study findings. For deaths in hospitals, the validation characteristics (concordance, sensitivity, and positive predictive value) of registration diagnoses were derived for leading causes of death, using the MR diagnoses as the reference standard. The study findings were subsequently applied to estimate cause-specific mortality patterns for all hospital deaths in the registration data. In a subsample of hospital deaths for which a VA diagnosis was also available, validation characteristics were derived for VA methods (the VA validation study) [6,7].

A verbal autopsy was completed for 6,328 deaths outside health facilities. These VA diagnoses were compared with VR diagnoses for the same deaths. Patterns of misclassification between VR and VA diagnoses were assessed, and *kappa *measures of agreement were used to determine reliability of registration diagnoses because no reference diagnoses ("gold standards") are available to measure validity for these deaths. The VA diagnoses were then used to estimate cause-specific mortality proportions in the study sample in view of the more rigorous methods applied compared to registration diagnoses. Subsequently, findings from the VA validation study conducted on hospital deaths were used to adjust the VA-based cause-specific mortality proportions for these 6,328 deaths, assuming that the validation characteristics of VA for hospital deaths would be the same for deaths outside hospitals. These adjusted VA diagnostic distributions were then used to estimate cause-specific mortality patterns for deaths outside health facilities in Thailand.

Finally, mortality estimates from the two arms of the study were summed and adjusted to fit the overall numbers of deaths by age and sex estimated from demographic analysis to derive detailed cause-specific mortality estimates for Thailand in 2005 [8].

## Results

Table 1 shows the distribution of the study sample across provinces as well as the results from data collection for the two broad categories of deaths in hospitals and deaths elsewhere. Losses to follow up were higher for deaths in hospitals, including deaths for which households could not be traced to obtain consent for participation in the study (397 deaths), as well as deaths for which consent was obtained but adequate medical records were not available (931 deaths). While the loss of some of the latter deaths with inadequate medical records could have been due to the limited time period between admission and death, these findings suggest that urgent measures are needed to improve the quality of medical records in hospitals in Thailand. Overall, about 29% of hospital deaths in the sample were lost to follow up.

**Table 1:** Distribution of study sample and results of data collection by province and place of death in Thailand, 2005

				Data collection							
				
				Hospital deaths					Home deaths		
				
Region (% deaths)	Province	Study districts	Study Sample^a^	Only MR ^b^	MR & VA	Only VA ^c^	Lost to follow up ^d^	Total	VA	Lost to follow up ^e^	Total
North East (32.4%)	Ubolrajthani	7	2699	81	525	117	24	747	1812	140	1952
	Leoi	3	1040	22	165	97	17	301	690	49	739

North (22.5%)	Chiang Rai	3	1849	73	404	160	55	692	972	185	1157
	Payow	3	751	20	136	73	18	247	435	69	504

Central (24.5%)	Supanburi	3	1970	68	472	192	54	786	1087	97	1184
	Nakhon Nayok	2	866	9	188	118	29	344	450	72	522

South (12.6%)	Songkhla	2	1059	140	277	43	37	497	430	132	562
	Chumpon	2	400	5	111	47	8	171	200	29	229

Bangkok Metropolitan Area (7.5%)		3	1350	340	280	84	155	859	252	239	491

Total		28	11984	758	2558	931	397	4644	6328	1012	7340

^a^Sampling plan included 15% oversampling for outer regions and 50% for Bangkok

^b^Deaths for which adequate medical records (MR) traced but refused verbal autopsy (VA)

^c^Deaths for which medical records either not traced or of inadequate quality to derive causes of death

^d^Households not located due to incorrect address in registration records, or migration

^e^Deaths for which household not located or refused VA

About half as many (1,012 cases, or 14%) home deaths were lost to follow up. Household contact and VA response rates were particularly high in the study provinces located in the Northeast region, but only around 50% in Bangkok, with loss to follow up arising roughly equally from inability to trace households and from refusal to participate in VA interviews. While these findings suggest the broad acceptance of VA by the community, they nonetheless indicate the need for more adequate sensitization about its public health utility, particularly in urban areas.

It is important to consider the extent to which such losses to follow up, for whatever reason, might lead to serious distortions in the proportionate distribution by cause of the sample, and hence affect the generalizability of the results. Table 2 shows the proportionate distributions by registered cause as derived from: the sampling frame (i.e., national death registration data); the sample drawn for the study; and the sample of deaths eventually recruited and used for mortality estimation, comprising 3,316 hospital deaths and 6,328 deaths outside hospitals.

**Table 2:** Proportionate mortality distributions (in %) for leading causes of death in Thailand,2005: vital registration data, selected study sample and final study recruited sample

Cause	ICD codes	Registration data	Study sample	Recruited sample
Symptoms signs and ill defined conditions	R00-R99	38.2	37.5	39.6
Septicaemia	A40-A41	5.8	5.7	5.4
All other external causes	W20-W64, W75-W99, X10-X39, X50-X59, Y10-Y89	4.8	4.7	4.2
Other malignant neoplasms	*	4.0	4.0	4.0
Cerebrovascular diseases	I60-I69	4.0	4.1	3.8
Genitourinary diseases	N17-N98	3.2	3.7	3.9
Liver cancer	C22	3.2	2.7	2.9
Pneumonia	J12-J18	3.1	3.0	2.8
Ischaemic heart disease	I20-I25	2.9	3.1	2.8
Transport accidents	V01-V99	2.8	2.9	2.8
Diseases of the liver	K70-K76	2.1	2.0	2.0
Lung cancer	C33-C34	2.0	2.1	2.1
HIV/AIDS	B20-B24	2.0	2.0	1.8
Diabetes mellitus	E10-E14	1.9	1.9	1.9
Other respiratory diseases	J00-J06, J30-J39, J60-J98	1.8	1.9	1.9
Other heart diseases	I26-I51	1.5	1.6	1.5
Chronic lower respiratory diseases	J40-J47	1.4	1.7	1.6
Tuberculosis	A15-A16	1.3	1.2	1.2
Other diseases of the nervous system	G04-G25, G31-G98	1.3	1.3	1.3
Other digestive disorders	K00-K22, K28-K66, K80-K92	1.2	1.1	1.1
All other causes	All other codes	11.5	11.8	11.4
Total deaths (100%)		**395374**	**11984**	**9644**

*C17, C23-C24, C26-C31, C37-C41, C44-C49, C51-C52, C57-C60, C62-C66, C68-C69, C73-C81, C88, C96-C97

There is clearly a very close approximation of the proportionate distributions of registered causes in the sampling frame, the study sample, and in the registered causes for the recruited sample. This finding indicates that any effects resulting from the sampling procedure or from losses to follow up are unlikely to greatly affect the generalizability of study findings.

**Figure 4:**
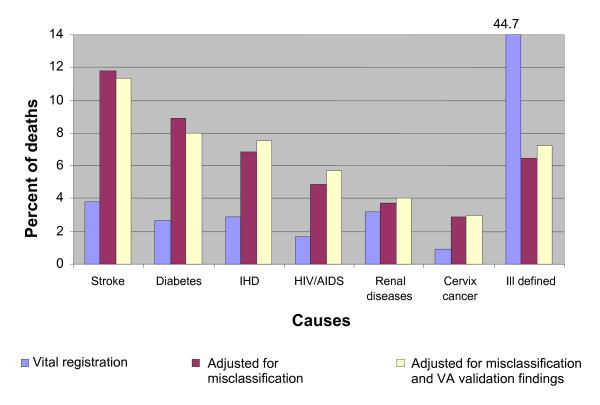
**Proportionate mortality (in %) for selected leading causes of death based on study findings, for females, Thailand, 2005**.

**Figure 5:**
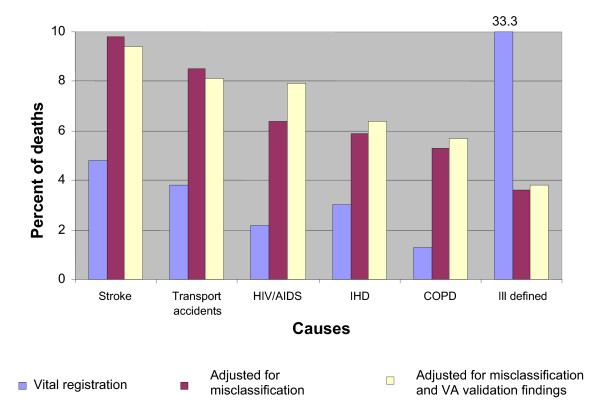
**Proportionate mortality (in %) for selected leading causes of death based on study findings, for males, Thailand, 2005**.

Figures 4 and 5 provide a summary of the study findings in terms of estimated proportionate mortality from leading causes of death in males and females, respectively. The reassessment of causes of death via medical records review or verbal autopsy resulted in a substantially different broad cause-of-death distribution than that suggested by the vital registration system. In particular, these methods led to a massive reduction in the percentage of deaths assigned to ill-defined causes for both males and females, declining from 33% to 45% of all deaths in vital registration to 4% to 6%, with the vast majority of these deaths being attributed to specific causes of death from our methods. Adjustment for the systematic undercounting or overcounting of specific causes by the VA procedure (shown in the bar titled "adjusted" in Figures 4 and 5) resulted in important changes to estimated proportionate mortality, notably from HIV/AIDS, ischemic heart disease, and diabetes.

Interestingly, in addition to cardiovascular diseases and cancers, the study identified diabetes, renal diseases, and chronic obstructive pulmonary disease as important noncommunicable diseases, the magnitude of mortality from which was substantially less evident from registration data. Further, many injury deaths in the study sample with nonspecific causes were reallocated to specific external causes upon verification, highlighting suicide, assault, and drowning as external causes of public health importance, especially among males, in addition to transport accidents.

## Discussion

Despite the fundamental importance of cause-of-death information for health planning, few countries regularly evaluate the quality of their cause-of-death statistics or the functioning of the civil registration system that routinely generates them. This study has conducted a systematic evaluation of registration data, the results of which have yielded corrected estimates of the true underlying cause-of-death pattern.

The study has some important limitations, both in terms of the methods used for data collection and the generalizability of the study findings to derive final mortality estimates for Thailand. While these have been discussed in other manuscripts in this series, in general, standard verbal autopsy and medical records review procedures to verify registered causes of death have been successfully applied elsewhere [24,26]. In the absence of widespread autopsy, we believe that careful medical records review provides a reasonable basis for ascertaining the true underlying cause of death in hospitals. We believe that the application of the findings from VA validation to adjust for systematic biases resulting from the use of VA strengthens the empirical basis for estimating population-level cause-specific mortality patterns from the study. More importantly, we conclude that rigorous and careful application of VA methods can drastically improve the information content of cause-of-death data at comparatively low cost.

We further explore these research questions, methods, and results in companion papers that provide more detailed insights into the validity and reliability of cause-of-death statistics in Thailand and the likely pattern of age- and cause-specific mortality rates, taking into account estimated levels of underreporting of deaths and the misclassification patterns that this study has identified [6,8]. Collectively, this research will greatly strengthen the evidence base for health policy in Thailand. The collateral benefits of this study lie in the development of scientific methods for such research, as well as the strengthening of technical capacity within the Thai Ministry of Public Health for the conduct of evaluation research and its integration into routine cause-of-death data collection systems. To our knowledge, no comparable study of the reliability of cause-of-death data has been attempted in a developing country. This research could serve as a model for similar necessary investigations into the quality of mortality statistics in other developing countries.

## Competing interests

The authors declare that they have no competing interests.

## Authors' contributions

ADL and YP conceived the study. CR designed the study in conjunction with ADL and YP. YP, JP, WP, and NS coordinated and undertook the field work. CR, YP, JP, WP, and NS undertook analysis. CR drafted the manuscript. ADL and YP assisted with interpretation of findings and finalizing the manuscript. All authors read and approved the final version.
